# IL-33/ST2 immunobiology in coronary artery disease: A systematic review and meta-analysis

**DOI:** 10.3389/fcvm.2022.990007

**Published:** 2022-10-20

**Authors:** Renli Liu, Liping Liu, Chaojie Wei, Dong Li

**Affiliations:** Department of Immunology, College of Basic Medical Sciences, Jilin University, Changchun, China

**Keywords:** IL-33/ST2, gene, SNPs, CAD, immunobiology

## Abstract

The IL-33/ST2 axis is reported to be controversially associated with coronary artery disease (CAD). A systematic review of the association between the IL-33/ST2 axis and CAD revealed that IL-33/ST2 plays a protective role in CAD and serum sST2 and IL-33 levels are increased in patients with cardiovascular disease. Therefore, the association of IL-33/ST2 single nucleotide polymorphisms (SNPs) with CAD prevalence, prognosis, and risk factors was assessed by performing a meta-analysis. Through a literature search of relevant articles in various databases using the relevant keywords, seven studies were included in the analysis. The meta-analysis showed that the IL-33/ST2 axis was associated with increased CAD risk [pooled odds ratio (OR) = 1.17, 95% confidence interval (CI): 1.13–1.20]. Gene subgroup analysis showed a close association of *IL1RL1* (OR = 1.25, 95% CI: 1.20–1.30; *I*^2^ = 85.9%; *p* = 0.000) and *IL1RAcP* (OR = 1.42, 95% CI: 1.26–1.60; *I*^2^ = 27.1%; *p* = 0.203) with increased CAD risk. However, the association for the *IL-33* gene was not statistically significant. SNPs rs7044343 (T), rs10435816 (G), rs11792633 (C) in *IL-33* gene were associated with a protective effect in CAD. However, rs7025417 (T) in *IL-33*, rs11685424 (G) in *IL1RL1*, rs950880 (A) in *sST2*, and rs4624606 (A) in *IL1RAcP* were related to increased CAD risk. Overall, polymorphisms in IL-33/ST2 axis components were associated with increased CAD risk. These results may help identify key features of IL-33/ST2 immunobiology in CAD along with potential treatment strategies to lower disease burden.

## Introduction

Interleukin-33 (IL-33), a member of the IL-1 cytokine superfamily, is an important regulator of pathological inflammation, immune homeostasis, fibrosis, and repair processes (1). The IL-33 receptors ST2L and sST2 are constitutively expressed in cells of the cardiovascular system, especially endothelial cells (2). Coronary artery disease (CAD) is caused by complex chronic inflammatory processes facilitated by the innate and acquired immune systems. Many cytokines are involved in CAD development (3). The IL-33/ST2 axis is involved in diverse areas of cardiovascular disease. IL-33 plays a role in cardiovascular disease either at the genetic level through regulation of transcription or as a classically active IL-33 functions that acts as an “alarmin” or cytokine. IL-33, upon interaction with ST2L, has been reported to prevent myocardial apoptosis, and alleviate myocardial fibrosis and myocardial hypertrophy (4). Thus, it inhibits the progression of atherosclerosis. However, clinically, serum IL-33 levels are elevated in patients with cardiac failure and stent restenosis after myocardial infarction (5). Further, sST2 is a biomarker of disease severity and prognosis for most cardiovascular diseases. Many studies have suggested that sST2 attenuates the cellular and beneficial actions of IL-33 in the cardiovascular system (6). However, there are no actual studies to prove that sST2 is harmful in CAD. Therefore, the theoretically beneficial effect of the IL-33/ST2 axis on the cardiovascular system is not reflected in practice.

Only a few recent studies on genetic linkage have attempted to explain the paradoxical relation between IL-33/ST2 and CAD (7, 8). Therefore, this article describes the known immunobiological functions of the IL-33/ST2 pathway in CAD occurrence and development and also presents a meta-analysis of the IL-33/ST2 gene polymorphisms associated with an increased risk of CAD.

## Systematic review

### IL-33/ST2: Biological structure and function

**FIGURE 1:**
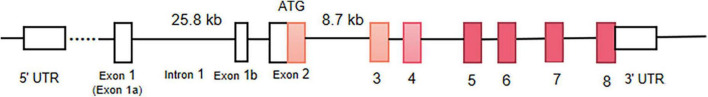
The human *IL-33* gene. The molecular weight of human IL-33 is about 18 kDa, and its gene span is > 42 kb. The main promoter of human IL-33 mRNA is upstream of the untranslated exon. The human *IL-33*-encoding gene contains eight exons. Exon 1b may replace the promoter. There are CAD related SNPs in promoter and intron 1 regions. Previous studies have reported the absence of splicing variants on exons 3/4/5/8, which may lead to the production of spliced IL-33 proteins.

**FIGURE 2:**
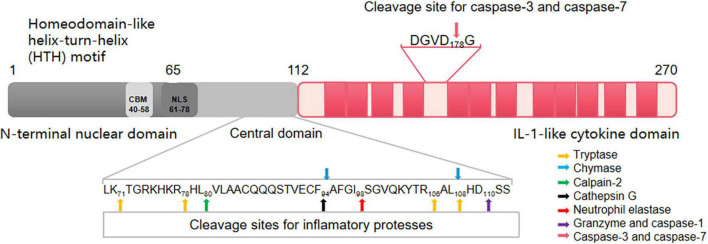
The human IL-33 protein. Human IL-33 consists of 270 amino acids (aa). The protein includes the N-terminal nuclear localization signal, C-terminal IL-1-like cytokine domain, and intermediate divergence. The homeodomain-like helix-turn-helix (HTH) motif is constituted of aa 1–65 in the N-terminal region. Aa 40–58 in the N-terminal region of IL-33 form the chromatin-binding motif (CBM). CBM mediates IL-33 to bind to chromatin in a protein-protein interaction. The aa at positions 65–78 form the nuclear localization signal (NLS). Notably, CBM and NLS are critical for the nuclear localization of IL-33. Human mature IL-33 residues aa 112–270 (mouse mIL-33 residues aa 109–266). Many cleavage sites for inflammatory proteases (including chymase, tryptase, neutrophil elastase, cathepsin G, and elastase process) are found in the central domain of IL-33. These inflammatory proteases cleave full-length IL-33 in the central domain and produce cytokines with a more bioactive mature. Amino acids at position 111 of IL-33 is inactivated by caspase-1. Granzyme B processes full-length IL-33 into 18-kDa form which corresponds to IL-33_111–270_. The IL-1-like cytokine domain at aa 112–270 is the C-terminal region that is structurally homologous to other cytokines of the IL-1 family (IL-1α, IL-1β, and IL-18). After being digested by caspase-3 and caspase-7 at aa 178, IL-33 is often released as a mature cytokine that subsequently binds to its receptor, ST2. Different parts of the IL-33 sequence control different aspects of its biology, including subcellular localization, exocrine secretion, and functional maturation. Based on these sequence signals, IL-33 plays a dual role as a cytokine that activates downstream signals when released from necrotic cells and as a nuclear factor that contains transcriptional regulation in cells.

The human IL-33 gene is present on chromosome 9p24.1. IL-33 is known to bind chromatin through the H2A-H2B histone complex (9), which alters the interaction between nucleosomes to regulate the degree of chromosome compression, and thus alters the transcription of target genes (Figure 1). The IL-33 protein possesses an N-terminal nuclear domain, a C-terminal IL-1-like cytokine domain, and a homeodomain-like helix-turn-helix (HTH) motif at the divergence part in the middle of its sequence (10). The chromatin-binding motif (CBM, aa 40–58) at its N-terminal binds to chromatin through protein-protein interactions and co-determines the nuclear targeting of IL-33 through a predicted bipartite nuclear localization sequence (NLS, aa 65–78) (9) (Figure 2). The precursor of IL-33 (proIL-33) normally resides in the nucleus. When cells are stimulated by mechanical stress, inflammatory cytokines, or necrosis, extracellular proteases process proIL-33 such that it can act in an autocrine/paracrine manner as an “alarmin” on neighboring cells or on various ST2 receptor-expressing immune cells (11). This complex biological structure and function of IL-33 is an important reason for the multiple roles of IL-33 in CAD. For example, SNPs of IL-33 in different cellular localization fragments have different or even opposite roles in the pathogenesis of CAD.

The human *IL1RL1* gene is present on chromosome 2q12 (12). IL1RL1 (ST2) has four transcript isoforms, of which the most important are ST2L (IL1RL1-b) and sST2 (IL1RL1-a). The *IL1RL1* gene encodes both ST2L and sST2 through alternative promoter activation. Two promoters at the proximal and distal parts of the *ST2* gene regulate *ST2* gene transcription. There are two SNPs in the distal parts are related to the susceptibility of CAD. IL-33 can self-regulate both ST2L and sST2 mRNA transcription (13). The link between the gene structure of *IL-33* and its receptor *IL1RL1* and CAD determines the important role of IL-33/ST2 pathway in CAD disease.

### IL-33/ST2 and coronary artery disease

**FIGURE 3:**
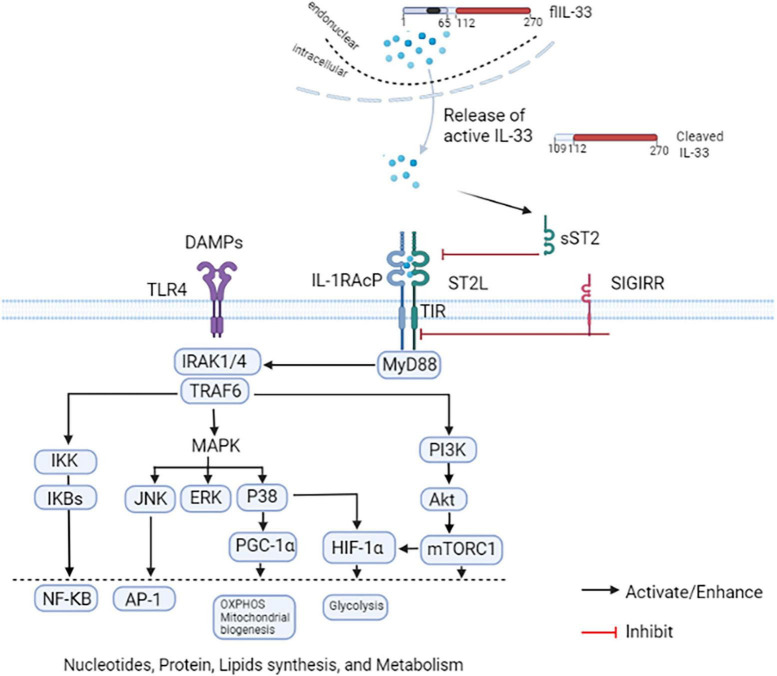
IL-33/ST2 axis. IL-33 is normally localized in the cell nucleus. The full-length (fl) IL-33 protein is released in the endoplasmic reticulum-Golgi independent but ATP-dependent pathway during cell damage or necrosis. The flIL-33 is then cleaved by inflammatory proteases into a form with increased activity. A heterodimeric receptor complex is formed by IL-33 and ST2L with IL-1R accessory protein (IL-1RAcP) The complex activates the downstream MyD88 pathway, and then activates NF-κB, AP-1, PGC-1α, and HIF-1α to promote gene, protein, and lipid synthesis as well as cellular metabolism. A single IL-1-associated receptor SIGIRR negatively regulates the IL-33/ST2 axis. NF-κB and AP-1 are involved in the pathogenesis of various diseases by promoting inflammatory responses and inhibiting apoptosis. Activation of P38 can promote PGC-1α, OXPHOS, and mitochondrial fission and fusion; it can also lead to the activation of HIF-1α and enhancement of glycolysis. mTORC1 can also enhance HIF-1α expression and glycolysis enhancement in monocytes/macrophages.

IL-33, a member of the IL-1 family of cytokines, is the ligand of the orphan receptor for ST2 (IL1RL1) (1). ST2 undergoes differential splicing to form the following subtypes: a transmembrane ligand (ST2L), a soluble component (sST2), and ST2V (13). ST2L is a transmembrane receptor; sST2 is a soluble receptor existing in blood circulation; and ST2V is a new variant of IL-33. ST2L and sST2 in the circulatory system are majorly found in the endothelial cells of the aorta and coronary arteries as well as immune cells such as T cells (2). The detailed biological structure and function of IL-33 have been described previously. IL-33 combined with chromatin is mainly stored in endothelial and epithelial cells nuclear. Then, it is released extracellular as a cytokine (14). IL-33 and ST2L bind to inflammatory cell membranes, which trigger intracellular signaling cascades that are enhanced by histones. A heterodimeric receptor complex is formed by IL-33 and ST2L with IL-1R accessory protein (IL-1RAcP) (15). This heterodimeric receptor complex utilizes the Toll/IL-1 receptor domain of IL-1RAcP for recruiting IRAK1, IRAK4, TRAF6 and Myd88, and subsequently activates multiple signaling pathways such as IKK/NF-KB, MAPK/AP-1, and PI3K/mTORC1, thus promoting gene expression, protein and lipid synthesis, and cellular metabolism (16) (Figure 3). Further, extracellular IL-33 is reported to indirectly inhibit the MAPK/p38/NF-κB pathway activation in the heart following MI (17). IL-33 reduces proinflammatory responses by affecting the activation of these cytokines, thus becoming a potential therapeutic target for cardiac remodeling after MI.

**FIGURE 4:**
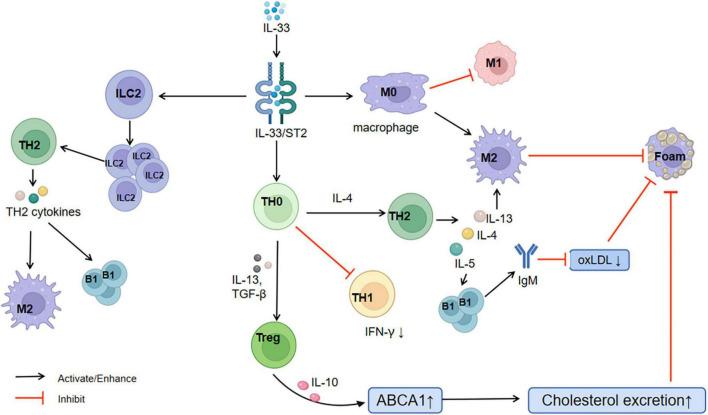
Relationship between IL-33/ST2 and CAD. CAD, coronary artery disease; M1, M1 phenotype of macrophages; M2, M2 phenotype of macrophages; TH1, T-helper 1; TH2, T-helper 2; ILC2, type 2 innate lymphoid cells; B1: B1 cells; Treg, regulatory T cells; Foam, foam cells; oxLDL, oxidized low-density lipoprotein; ABCA1, ATP-binding cassette transporter A1.

A major part of atherosclerosis is artery wall chronic inflammation (18). IL-33 binding to ST2L inhibits Th1 cytokine production (reduces IFN-γ levels) and significantly increases Th2 cytokine production (IL-4, IL-5, IL-6, IL-8, and IL-13) (19). Thus, IL-33/ST2L induces polarization of the anterior atherosclerotic immune response from the Th1 to the Th2 type and inhibits atherosclerosis progression (20). Simultaneously, IL-33/ST2L induces the amplification of type 2 innate lymphoid cells (ILC2s) and promotes their egress from the immune organ (21). ILC2s also activate the Th2 response (22) and release cytokines. The activated downstream cytokine IL-5 may stimulate the proliferation of B1 cells (23) and production of atheroprotective natural IgM antibodies, which inhibit oxidized low-density lipoprotein production by acting against the phosphorylcholine head group of the oxidized phospholipids within low-density lipoproteins (24). Th2 cytokines, especially IL-13, polarize macrophages into the M2 phenotype (25). This mechanism limits the formation of foamy cells and lipid accumulation within plaques. Together with IL-10, IL-33 elevates the ATP-binding cassette transporter A1 expression (26), promotes the excretion of human cholesterol (27), and inhibits the formation of macrophage foam cells (28). Regulatory T cells (Tregs) support metabolic function and adipocyte differentiation (29, 30). IL-33 can selectively amplify ST2^+^ Tregs. ST2^+^ Tregs promote IL-13 and TGF-β release to inhibit inflammation and CD4^+^ T cell proliferation (31, 32). Enhanced IL-10 production in macrophages promotes macrophage polarization to the M2 anti-inflammatory phenotype, which suppresses foam cell production (25) (Figure 4).

However, sST2 binds IL-33 reducing the amount of IL-33 that can bind to ST2L and altering the anti-atherosclerosis protective activity of IL-33 (33). During cellular stress, IL-33, ST2L, and sST2 are upregulated, and sST2 can be considered a regulatory mechanism for avoiding excessive activation of IL-33. Altara et al. (34) previously summarized the following reasons underlying increased sST2 levels during enhanced IL-33/ST2L responses: AP1 and NF-KB signaling can activate sST2-related promoters; and increased ST2L expression by increased endogenous IL-1β levels because the counter-regulated PI3K/AKT/mTOR signaling is inhibited by ST2L to decrease the levels of IL-1β. IL-33 promotes the activation of adhesion molecules (such as VCAM-1, ICAM-1, and E-selectin) and induces the expression of chemokines (such as CXCL1 and MCP-1) in human endothelial cells and activates inflammation, which can possibly enhance atherosclerotic lesion development in the vascular wall (18, 35). Clinically, serum IL-33 levels are elevated in patients with cardiac failure and stent restenosis after myocardial infarction (5, 36). However, the mechanisms of these paradoxical roles of IL-33/ST2 remain unclear.

## Relationship between single nucleotide polymorphisms in IL-33/ST2 and coronary artery disease: A meta-analysis

Considering the paradoxical role of the IL-33/ST2 axis on the cardiovascular system, we sought to explain the relationship between IL-33/ST2 and CAD based on the genetic linkage of the IL-33/ST2 axis. We thus performed a meta-analysis of all the studies related to CAD and the SNPs of IL-33/ST2 to identify the key features of the IL-33/ST2 immunobiology that have significance in CAD and the potential treatment strategies to lower disease burden. We searched for relevant articles published before April 1, 2022, without any restrictions on language. The following keywords and MeSH terms were used to search PubMed, Web of Science, Cochrane Library, and Clinicaltrials.gov databases: IL-33/ST2, Single Nucleotide Polymorphisms, Coronary Artery Diseases, nuclear protein, localization, metabolic, immunity, and Diseases.

### Study characteristics

**FIGURE 5:**
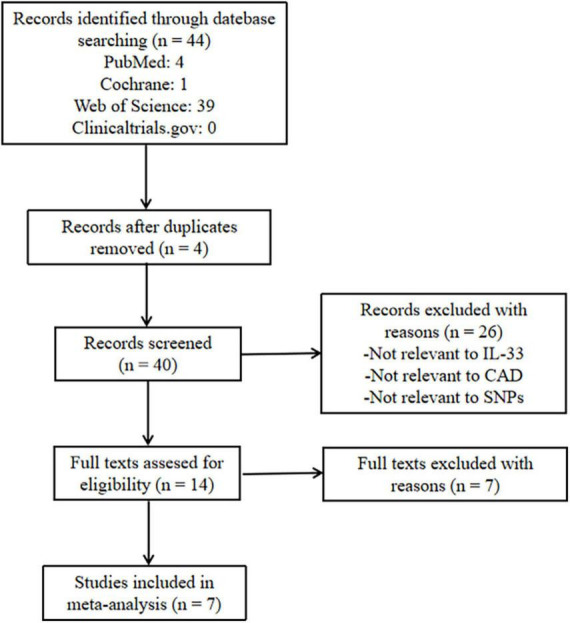
Flowchart of search strategy. CAD, coronary artery disease; SNP, single nucleotide polymorphism.

Figure 5 shows the literature-screening process. We first screened 44 studies, and after removing duplicates, selected the collected studies according to the following inclusion standards: (1) the study should be a case-control or clinical cohort study focusing on the link between IL-33/ST2 gene polymorphism and susceptibility to cardiovascular disease; (2) the study must provide adequate genotypic frequency information; (3) all cases must meet the diagnostic standard for cardiovascular disease; (4) the healthy controls should have their genotype frequencies in the Hardy-Weinberg equilibrium; (5) the studies should have a Newcastle-Ottawa scale score ≥ 5. Studies that did not meet these standards were excluded. Thus, seven case-control studies met our inclusion criteria for qualitative data analysis, and contained a total of 10686 cases and 10775 healthy subjects (7, 8, 37–41). Two researchers simultaneously screened the studies back-to-back and obtained consistent results. Detailed information on the finally included studies is shown in Table 1.

**TABLE 1:** Characteristics of the studies included.

References	Country	Ethnicity	Sample size		Genotyping method	SNP type (*gene*)	HWE test (*p*-value)
			Case	Control			
Tu et al. (37)	China	Asian	4,521	4,809	Rotor-Gene 6000 High-Resolution Melt (HRM) system	rs7025417(*IL-33*) rs11685424(*IL1RL1*)	>0.001
Wu et al. (8)	China	Asian	1,146	1,146	Sequenom MassARRAY and TaqMan assays	rs4624606(*IL-1RAcP*)	> 0.001
Yang et al. (38)	China	Asian	490	929	Sequenom MassARRAY and TaqMan assays	rs4624606(*IL-1RAcP*)	>0.05
Lin et al. (39)	China	Asian	532	601	TaqMan assays	rs950880(*IL1RL1*) rs13001325(*IL1RL1*)	>0.05
Angeles-Martínez et al. (40)	Mexico	Caucasian	1,095	1,118	TaqMan assays	rs7044343(*IL-33*)	>0.001
Nie et al. (41)	China	Asian	1,736	1,093	DNA sequencing analysis	rs7025417(*IL-33*)	>0.001
Li et al. (7)	China	Asian	1,166	1,079	Multiple Ligase Detection Reaction platform	rs7025417(*IL-33*) rs7044343(*IL-33*) rs10435816(*IL-33*) rs11792633(*IL-33*)	>0.05

SNP, single nucleotide polymorphism; HWE, Hardy-Weinberg equilibrium.

### Meta-analysis

**FIGURE 6:**
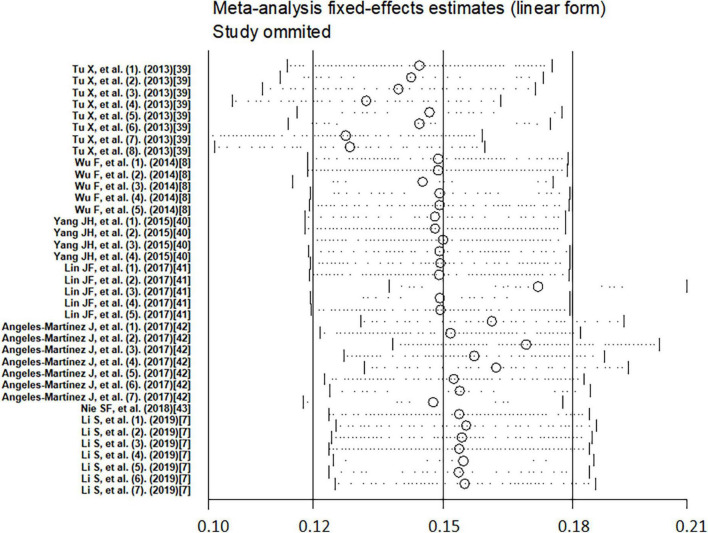
Meta-analysis of the relationship between IL-33/ST2 and coronary artery diseases risk OR, odds ratio; CI, confidence interval.

Figure 6 shows the multivariate-adjusted odds ratio (OR) for IL-33/ST2. The meta-analysis indicated that the IL-33/ST2 axis was significantly associated with increased CAD risk. The pooled OR was 1.17 (95% confidence interval (CI): 1.13–1.20), and high heterogeneity was observed (*I*^2^ = 88.2%; *p* < 0.001).

### Subgroup analysis

**FIGURE 7:**
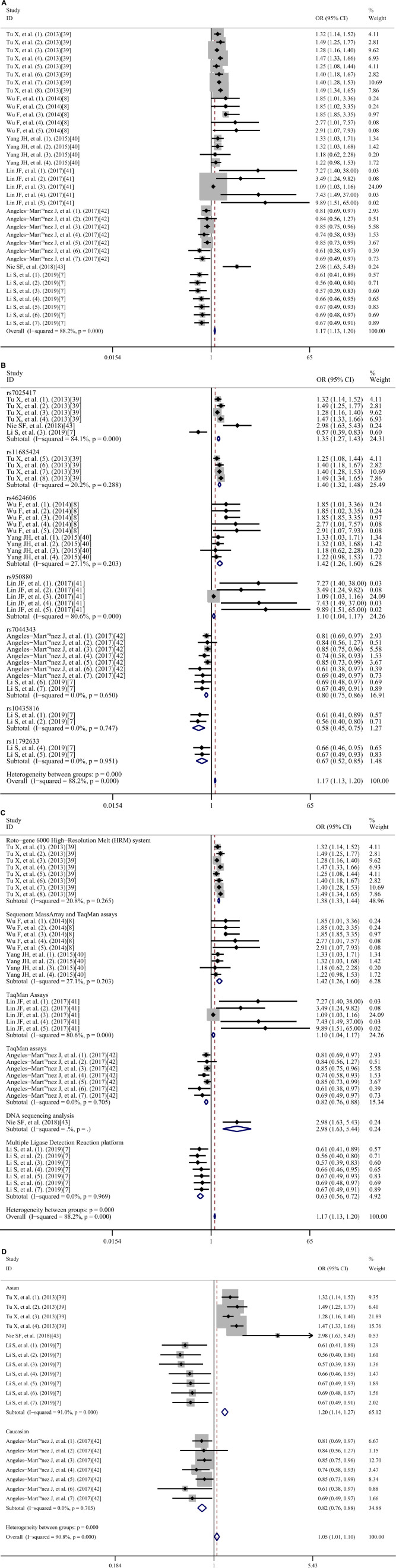
Subgroup analyses based on **(A)** genes, **(B)** single nucleotide polymorphisms, **(C)** genotyping methods, and **(D)** ethnicity. OR, odds ratio; CI, confidence interval.

The impact of potential factors on the individual risk of developing CAD was investigated using subgroup analysis (Figure 7). Gene subgroup analysis suggested that *IL1RL1* (OR = 1.25, 95% CI: 1.20–1.30; *I*^2^ = 85.9%; *p* = 0.000) and *IL1RAcP* (OR = 1.42, 95%CI: 1.26-1.60; *I*^2^ = 27.1%; *p* = 0.203) may be closely associated with increased CAD risk (Figure 7A). However, the association of the *IL-33* gene was not statistically significant, possibly because of polymorphisms. Analysis results of the SNP subgroups (Figure 7B) showed that rs7044343 (T) (OR = 0.80; 95% CI: 0.75–0.86; *I*^2^ = 0.0%; *p* = 0.650), rs10435816 (G) (OR = 0.58, 95% CI: 0.45–0.75; *I*^2^ = 0.0%; *p* = 0.747), and rs11792633 (C) (OR = 0.67, 95% CI: 0.52-0.85; *I*^2^ = 0.0%; *p* = 0.951) polymorphisms in the *IL-33* gene may be closely associated with a protective effect against CAD. However, rs7025417 (T) (OR = 1.35, 95% CI; 1.27–1.43; *I*^2^ = 84.1%; *p* = 0.000) in *IL-33*, rs11685424 (G) (OR = 1.40, 95% CI; 1.32–1.48; *I*^2^ = 20.2%; *p* = 0.288) in *IL1RL1*, rs950880 (A) (OR = 1.10, 95% CI; 1.04–1.17; I^2^ = 80.6%; *p* = 0.000) in *sST2*, and rs4624606 (A) (OR = 1.42, 95% CI: 1.26–1.60; *I*^2^ = 27.1%; *p* = 0.203) in *IL1RAcP* may be closely associated with increased CAD risk. Subgroup analysis of the genotyping methods (Figure 7C) showed that the use of TaqMan assays and the Multiple Ligase Detection Reaction platform were negatively associated with increased CAD risk, whereas the others were positively associated with increased CAD risk. We also performed a subgroup analysis of ethnicity for IL-33 (7, 37, 40, 41) (Figure 7D), and found that Caucasian ethnicity may be closely associated with a protective effect against CAD, whereas Asian ethnicity was associated with increased CAD risk. The ethnicity of the study subjects for both IL1RL1 and IL1RAcP was Asian. All the OR values obtained were adjusted for risk factors associated with CAD, including age, smoking, drinking, sex, triglyceride levels, body mass index, diabetes mellitus, hypertension, and a family history of CAD.

### Meta-regression

**FIGURE 8:**
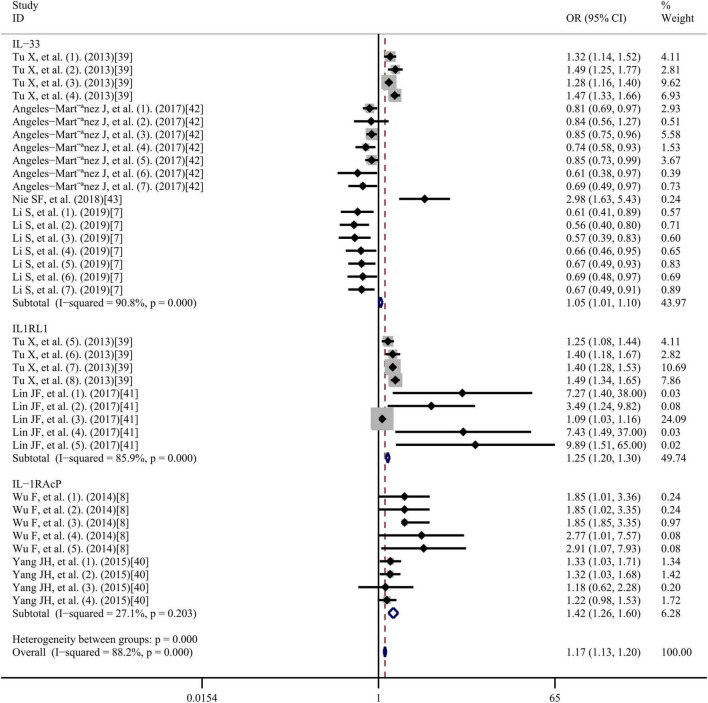
Sensitivity analysis of the association between IL-33/ST2 and coronary artery disease.

**FIGURE 9:**
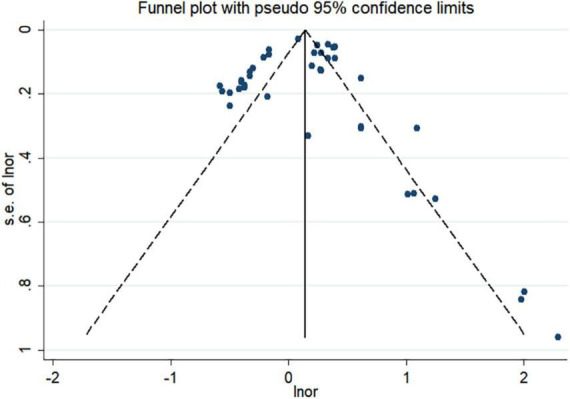
Funnel plot of the association between IL-33/ST2 and coronary artery disease. s.e, standard error; lnor, logarithm of the odds ratio.

To investigate the source of heterogeneity, we performed meta-regression using genes, SNPs, genotyping methods, alleles, and ethnicity as covariates (Table 2). The results showed that genes, SNPs, alleles, and ethnicity are the main sources of possible heterogeneity in the association of IL-33/ST2 genetic polymorphisms with CAD susceptibility. The other covariates had no significant effect on inter-study heterogeneity. The sensitivity analysis results revealed that no study significantly affected the overall pooled OR (Figure 8). The funnel plot was used for assessing publication bias (Figure 9), Begg test, and Egger test; however, no publication bias was found (Begg test *p* = 0.349; Egger test *p* = 0.213).

**TABLE 2:** Meta-regression based on genes, SNPs, genotyping method, and alleles as covariates of coronary artery disease.

Covariate	*p*
Gene	0.001
SNPs	0.001
Genotyping method	0.207
Alleles	0.048
Ethnicity	0.028

SNP, single nucleotide polymorphism.

## Discussion

The beneficial and harmful effects of the IL-33/ST2 axis in patients with CAD are debatable (34, 42–44). Although several studies have reported the role of the IL-33/ST2 axis in CAD from a genetic perspective, the results remain controversial. In this study, we performed a meta-analysis to quantify this aspect based on previously published studies. Our meta-analysis results indicated that the IL-33/ST2 axis was significantly associated with increased CAD risk, consistent with the findings targeting IL1RL1 and IL1RaCP.

Among the results of the subgroup analysis, several factors deserve consideration. First, the results were explained by the differences in the target genes of the IL-33/ST2 axis among the included study participants. The gene subgroup analysis results showed that IL1RL1 and IL1RAcP might be closely associated with increased CAD risk. However, the results for the *IL-33* gene were not statistically significant, possibly because of the genetic polymorphisms in IL-33; another possible explanation is that the expression levels of the *IL-33* gene may be affected by genetic variations in it, thereby altering its function. To date, no other genes in the IL-33/ST2 axis have been reported to be associated with CAD. The SNP subgroup analysis showed that the rs7044343 (T), rs10435816 (G), and rs11792633 (C) in *IL-33* may be closely related to CAD protection. A previous genetic study evaluated the potential influence of the *IL-33* rs3939286 polymorphism in the development of carotid intima media wall thickness (a surrogate marker of cardiovascular disease) in Caucasian patients with rheumatoid arthritis (RA) (a chronic inflammatory disease associated with atherosclerosis) (45). With respect to this, those patients with RA carrying the *IL-33* rs3939286 TT genotype exhibited lower carotid intima media wall values than those homozygous for the CC genotype. Also, patients with RA carrying the *IL-33* rs3939286 mutant T allele showed significantly lower carotid intima media wall thickness values than those carrying the wild allele C in this study. Interestingly, this potential effect conferred by *IL33* rs3939286 was independent of confounder factors (45). In contrast, rs7025417 (T) in *IL-33*, rs11685424 (G) in *IL1RL1*, rs950880 (A) in *sST2*, and rs4624606 (A) in *IL1RAcP* may be closely related with increased CAD risk. This dual role of IL-33 is closely related to the complex biological structure and function of IL-33. The mechanism of IL-33 in cardiovascular disease acts on the one hand at the genetic level through regulation of transcription, and also through a classically active IL-33 as an “alarmin” or cytokine. Different IL-33 segments have different cellular localization and different functions. As a nuclear factor plays a transcriptional regulatory role, as a cytokine processed by inflammatory proteases to a 10-fold expanded activity. The different IL-33 SNPs in our meta-analysis results have different or even opposite roles in the pathogenesis of CAD, consistent with the dual effects of IL-33 in the clinic.

Subgroup analysis of the genotyping methods showed that the use of TaqMan assays and Multiple Ligase Detection Reaction platform were negatively associated with increased CAD risk, whereas the others method was positively. However, no association was found between genotyping methods and gene polymorphisms. Age was a major risk factor for CVD, but all the ages of the included studies were clustered between 55 and 75, which were at high risk of cardiovascular disease, and the subgroup analysis of age was not too significant.

As heterogeneity is evident, we performed a subgroup analysis of the ethnicity for IL-33. Our results suggested that *IL-33* gene polymorphism is associated with increased CAD risk in Asians and strongly associated with CAD protection in Caucasians. The ethnicity of the subjects included in the studies on IL1RL1 and IL1RAcP was Asian and could not be classified. However, based on the ethnic typing of IL-33, ethnic differences could possibly be a source of inter-study heterogeneity regarding the role of *IL-33* gene polymorphisms in the pathogenesis of CAD. Natural selection and random genetic drift may be factors affecting individual genetic susceptibility.

Furthermore, related studies have identified obesity, diabetes, hypertension, serum sST2 levels, and GDF-15 levels as significant factors affecting the increased risk of CAD (39).

Heterogeneity is significant for meta-analysis and directly influences the interpretation of its results. Therefore, exploring the potential sources of heterogeneity is important for this study. Our meta-regression results showed that genes, SNPs, alleles, and ethnicity may be the major sources of heterogeneity in the association of IL-33/ST2 genetic polymorphisms with susceptibility to CAD. As raw data from the included studies were not included, the influence of other individual CAD risk factors such as sex could not be assessed with respect to the potential role of IL-33/ST2 gene polymorphisms in CAD development. Moreover, information on allele genotype frequencies was insufficient to be included in the study; thus, allele genotype frequencies may also be a cause of heterogeneity. Although this study has many limitations, this study is among the first meta-analysis to focus on the association between IL-33/ST2 and CAD risk from the perspective of gene polymorphisms. The sensitivity analysis results reveal that no single study significantly affected the overall pooled OR. Further, no publication bias was found based on the funnel plot, Begg test, and Egger test.

## Conclusion

Our meta-analysis indicated that the IL-33/ST2 axis was significantly associated with increased CAD risk. This is consistent with the findings targeting both IL1RL1 and IL1RaCP. The SNPs rs7044343 (T), rs10435816 (G), and rs11792633 (C) in the *IL-33* gene of individuals with Asian ethnicity may be closely related to the protective effect of CAD. Further, rs7025417 (T) in the *IL-33* gene, rs11685424 (G) in *IL1RL1*, rs950880 (A) in *sST2*, and rs4624606 (A) in *IL1RAcP* in individuals of Asian ethnicity, may be closely associated with increased CAD risk. Thus, IL-33/ST2 gene polymorphisms may function as potentially useful biomarkers for the early diagnosis of CAD. However, owing to the abovementioned limitations, further studies with large sample sizes are required for obtaining a more representative statistical analysis. Overall, these results may help identify the key features of the IL-33/ST2 immunobiology in CAD and facilitate the development of potential treatment strategies to lower disease burden.

## Data availability statement

The original contributions presented in the study are included in the article/supplementary material, further inquiries can be directed to the corresponding author.

## Author contributions

RL: conceiving the research, data analysis, and manuscript writing. LL: data analysis. CW: screening of studies. DL: manuscript review. All authors have read and agreed to the published version of the manuscript.
